# Comparison of cerebral metabolic rate of oxygen, blood flow, and bispectral index under general anesthesia

**DOI:** 10.1117/1.NPh.10.1.015006

**Published:** 2023-03-06

**Authors:** Susanna Tagliabue, Claus Lindner, Ivette Chochron da Prat, Angela Sanchez-Guerrero, Isabel Serra, Michał Kacprzak, Federica Maruccia, Olga Martinez Silva, Udo M. Weigel, Miriam de Nadal, Maria A. Poca, Turgut Durduran

**Affiliations:** a ICFO – Institut de Ciències Fotòniques, The Barcelona Institute of Science and Technology, Barcelona, Spain; b Vall d’Hebron University Hospital, Department of Anesthesiology, Barcelona, Spain; c Vall d’Hebron University Hospital Research Institute, Neurotraumatology and Neurosurgery Research Unit, Barcelona, Spain; d Centre de Recerca Matemàtica, Bellaterra, Spain; e Barcelona Supercomputing Center—Centre Nacional de Supercomputació, Spain; f Nalecz Institute of Biocybernetics and Biomedical Engineering PAS, Warsaw, Poland; g HemoPhotonics S.L., Mediterranean Technology Park, Barcelona, Spain; h Universidad Autònoma de Barcelona, Plaça Cívica, Barcelona, Spain; i Vall d’Hebron University Hospital, Department of Neurosurgery, Barcelona, Spain; j Institució Catalana de Recerca i Estudis Avançats, Barcelona, Spain

**Keywords:** diffuse correlation spectroscopy, time-resolved spectroscopy, propofol-induced anesthesia, bispectral index, diffuse optics, cerebral blood flow, cerebral metabolic rate of oxygen, cerebral physiological changes, near-infrared spectroscopy, diffuse optics

## Abstract

**Significance:**

The optical measurement of cerebral oxygen metabolism was evaluated.

**Aim:**

Compare optically derived cerebral signals to the electroencephalographic bispectral index (BIS) sensors to monitor propofol-induced anesthesia during surgery.

**Approach:**

Relative cerebral metabolic rate of oxygen (${\mathrm{rCMRO}}_{2}$) and blood flow (rCBF) were measured by time-resolved and diffuse correlation spectroscopies. Changes were tested against the relative BIS (rBIS) ones. The synchronism in the changes was also assessed by the R-Pearson correlation.

**Results:**

In 23 measurements, optically derived signals showed significant changes in agreement with rBIS: during propofol induction, rBIS decreased by 67% [interquartile ranges (IQR) 62% to 71%], ${\mathrm{rCMRO}}_{2}$ by 33% (IQR 18% to 46%), and rCBF by 28% (IQR 10% to 37%). During recovery, a significant increase was observed for rBIS (48%, IQR 38% to 55%), ${\mathrm{rCMRO}}_{2}$ (29%, IQR 17% to 39%), and rCBF (30%, IQR 10% to 44%). The significance and direction of the changes subject-by-subject were tested: the coupling between the rBIS, ${\mathrm{rCMRO}}_{2}$, and rCBF was witnessed in the majority of the cases (14/18 and 12/18 for rCBF and 19/21 and 13/18 for ${\mathrm{rCMRO}}_{2}$ in the initial and final part, respectively). These changes were also correlated in time (R>0.69 to R=1, p-values<0.05).

**Conclusions:**

Optics can reliably monitor ${\mathrm{rCMRO}}_{2}$ in such conditions.

## 1 Introduction

Anesthetic agents suppress the patient’s awareness by disrupting the neuronal activity and by preventing the formation of memories in a dose-dependent manner.^1^ This work focuses on the anesthetic effect of propofol, which is a non-barbiturate intravenous agent used commonly for the induction and maintenance of anesthesia and for sedation in critical care.^2^^,^^3^ Of particular interest, due to propofol effect on the central nervous system, propofol induces a decrease in cerebral metabolic rate of oxygen (${\mathrm{CMRO}}_{2}$),^4^^,^^5^ and therefore, a dose-dependent depression of cerebral blood flow (CBF) while maintaining the physiological coupling between the two.^4^^,^^6^^–^^9^

The proper use of anesthesia is a complex procedure. One aspect, as demonstrated by many studies, is the need to record the state of consciousness during surgical procedures.^10^^–^^18^ This is done by the continuous monitoring of the effect of anesthetic drugs on the brain, in particular, by electroencephalographic (EEG) methods that monitor cerebral electrophysiology. A popular parameter is the so-called bispectral index (BIS) (former Covidien, now Medtronic plc, Ireland), which is a processed EEG measure that summarizes brain activity and is considered as a measure of the depth-of-anesthesia.^19^^–^^21^ The BIS algorithm was developed based on adult EEG data and combines several readings of time, frequency, and high-order spectral sub-components into a single dimensionless parameter that scales from 0 (cortical electrical silence) to 100 (awake) with increasing brain activity.^14^ Awake, unsedated individuals typically have BIS values >97, while the BIS value is progressively reduced by drug-induced sedation,^22^ BIS ranges between 40 and 60 for anesthetic maintenance^23^ during general anesthesia. It is assumed that the BIS provides real-time feedback on consciousness during surgical procedures^19^^,^^24^^–^^26^ and is widely used to obtain real-time pharmacodynamic information during propofol-induced anesthesia.^14^^,^^19^^,^^27^^–^^33^ Moreover, BIS is often considered a surrogate indicator for metabolism.^14^^,^^28^^,^^29^^,^^34^

However, BIS only may not be sufficient to provide a full picture of the brain under anesthesia,^14^^,^^35^^–^^40^ especially during the period of recovery to awareness.^38^ In the long run, a better understanding of the relationship of BIS and the cerebral hemodynamics and oxygen metabolism may be helpful to consider optical neuro-monitoring as a means to complement BIS. This may be a way for improved reliability and a way for improved safety procedures to minimize brain injury during anesthesia.

Techniques making the use of photon diffusion using near-infrared light have the potential to aid in this matter and are a topic for ongoing research.^10^^,^^15^^–^^18^^,^^41^^–^^46^ In fact, the combination of near-infrared time-resolved spectroscopy (TRS) and diffuse correlation spectroscopies (DCS), also called hybrid diffuse optics (DO), can non-invasively measure microvascular blood oxygen saturation (${\mathrm{StO}}_{2}$) and microvascular blood flow index (BFI).^47^ This together with the knowledge of the readily available pulse oxygen saturation (${\mathrm{SpO}}_{2}$) allows one to estimate the oxygen extraction fraction (OEF) and the above-mentioned ${\mathrm{CMRO}}_{2}$.^48^^–^^51^ Both diffuse optical techniques have been employed in the investigation of the human brain’s autoregulatory mechanisms and hemodynamic properties *in vivo*.^52^^–^^55^

Some studies in the DO field have emphasized the importance of focusing on the moments of loss of conscience (LOC), caused by the injection of anesthetic, and recovery of conscience (ROC), after the vanishing of drugs.^10^^,^^16^^,^^17^ In fact, it is around these events that the greatest variations in the level of patient awareness are recorded. Consequently, these are the periods where the same is expected for the non-invasive hemodynamic signals.^10^^,^^15^^–^^17^

In the study presented here, the hybridization of TRS and DCS techniques sets out to show its capability of quantifying the oxygen metabolism and blood flow changes. The estimations of ${\mathrm{CMRO}}_{2}$ and CBF values are compared to electrical BIS readings, particularly in the moments of LOC and ROC aiming at validating optically derived ${\mathrm{CMRO}}_{2}$ as a surrogate index of metabolism.

## 2 Patients and Methods

A prospective study was conducted on patients above eighteen years of age, who underwent surgical procedures under standard general anesthesia using total venous anesthesia (TIVA) with propofol at Vall d’Hebron University Hospital (VHUH) from December 2014 to January 2016 and from June 2017 to January 2018. Patients were enrolled in the study based on the following inclusion criteria: (1) absence of a previous stroke, cerebral tumor, chronic hydrocephalus, neurodegenerative sickness, or evidence of carotid stenosis; (2) having no contraindications for TIVA with propofol; (3) being scheduled to undergo a general surgery, excluding laparoscopies with extreme patient positions, i.e., Trendelenburg position; (4) being in class I (healthy) or II (mild systemic disease) in the physical status classification system defined by the American Society of Anesthesiologists,^56^ and (5) having given written informed consent signed by the patient or next-of-kin. The study was approved by the VHUH Institutional Ethics Committee (protocol AC/U(AT)203/2012[3531]) and conducted in accordance with the Declaration of Helsinki.

### 2.1 Diffuse Optical Device

**Fig. 1:**
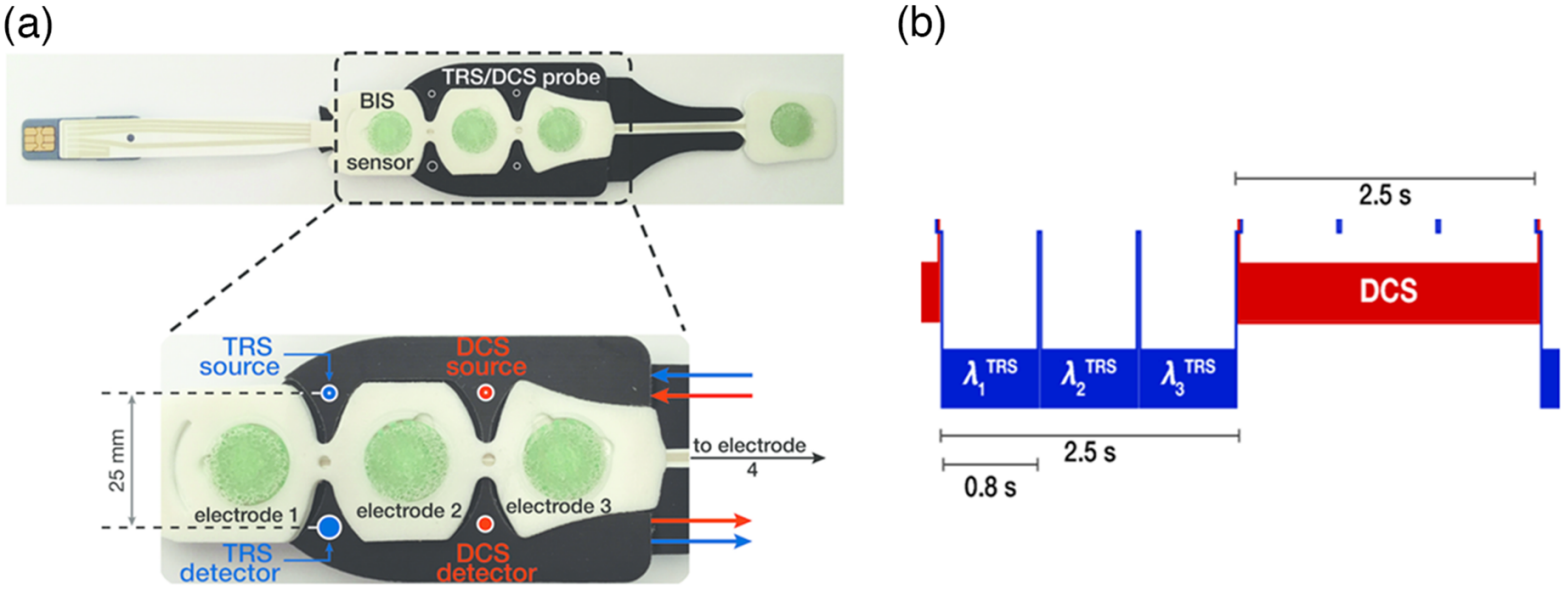
(a) Bottom view of the 3D printed combined BIS-TRS/DCS probe. The first three electrodes of the BIS sensor are placed on the subject’s left forehead followed by the black fiber pad which fits around the BIS sensor. The fourth electrode is attached next to the patient’s left eye. Source (top) and detector (bottom) fiber tips are placed in between the first and second electrodes, surrounded by TangoBlack shore 27 material. (b) TRS and DCS acquired data in alternation. The intensity autocorrelation curves were calculated for 2.5 s, while the system switched through all three TRS lasers with an acquisition time of 800-ms per wavelength.

The hybrid diffuse optical device used the combination of TRS and DCS and is described in detail in Refs. 57–59. Near-infrared light was sent into the tissue through a set of fibers incorporated into a combined DCS/TRS-BIS probe with a source-detector separation (ρ) of 25 mm for both optical modalities. Diffuse optical data were measured unilaterally on the left frontal lobe during extracranial surgical procedures due to the fact that the BIS signal was retrieved from this cerebral hemisphere. The combined DCS/TRS-BIS probe is shown in Fig. 1. The probe was designed around the BIS sensor while providing a compact housing for the source and detector fibers of both modalities, as shown in Fig. 1. It was 3D printed in TangoBlack shore 27 material (Stratasys Ltd., Eden Prairie, Minnesota) and incorporated in a head strap that was wrapped around the patient’s head. A Coban bandage (CobanTM, 3M TM) was used to keep stable the probe placement.

Briefly, the device consists of a single longitudinal mode laser operating at a wavelength of 785 nm for DCS measurements (DL785-120-SO, CrystaLaser, Reno, Nevada) and with pulsed light sources at 690, 785, and 830 nm for TRS measurements (BHLP-700, Becker & Hickl GmbH, Berlin, Germany). At the DCS detection site, a set of four single-photon counting avalanche photodiode collected the diffuse photons. A fiber bundle collected the TRS light and guided it into a hybrid photomultiplier detector, operated by a control card (HPM-100-50 and DCC-100 Becker & Hickl GmbH, Berlin, Germany). The detector signal was processed by a single photon counting card and a manufacturer-provided software (SPC-130 and SPCM, Becker & Hickl GmbH, Berlin, Germany).

The TRS and DCS modalities were multiplexed by an optical switch (Piezosystem Jena GmbH, Jena, Germany), so the DCS data could be acquired during 2.5 s, while a TRS measurement (800-ms per wavelength) was carried out alternately. Figure 1 shows the acquisition of TRS and DCS data. The entire device was controlled by a PC.

Events such as patient movement, substances injection, failures, etc. were recorded both by a marking routine included in the software, that recorded it in the data files of the hybrid device, and by detailed notes on measurement protocols, to be able to trace-back and relate such eventualities in the post-processing phase.

### 2.2 Clinical Data and Patient Medications

In addition to the recordings provided by the hybrid diffuse optical setup, ${\mathrm{SpO}}_{2}$, heart rate (HR) and mean arterial pressure (MAP) were extracted from either an anesthesia monitor (Datex-Ohmeda Aisys™, GE Healthcare, Little Chalfont, United Kingdom) by the open-source VitalSigns capture (VScapture) program^60^ or from a general monitor (Philips IntelliVue MX800, Koninklijke Philips N.V.). The BIS data were acquired by a BIS sensor (BIS Vista™, Medtronic plc, IRL). These signals were recorded by the same monitor with the same timestamps and were synchronized with the optical signals with a precision of 1 s and according to the beginning of the measurement.

General anesthesia was performed under total intravenous anesthesia with propofol at a concentration of 1% (Propofol Fresenius^®^, Fresenius Kabi Deutschland GmbH, Bad Homburg, Germany) using the Schneider model of target-controlled infusion anesthesia^61^ based on age, height, weight, and gender of the patient^3^^,^^62^ implemented in a TIVA system (Alaris Asena^®^ PK, Becton, Dickinson and Company, Franklin Lakes, New Jersey). After induction and tracheal intubation, pulmonary volume-controlled ventilation was maintained with a fraction of inspired oxygen (${\mathrm{FiO}}_{2}$) of 0.5, a tidal volume of 6 to 7  mg/kg, a respiration rate between 12 and 16 breaths per minute, a positive end-expiratory pressure of 4 to 6 mmHg, and a MAP between 60 and 80 mmHg.

It should be noted that the patients received further medications, such as fentanyl for analgesia,^63^^–^^65^ atropine sulfate, atracurium besylate, or rocuronium bromide to obtain neuromuscular blockade, remifentanil, neostigmine, lidocaine, and midazolam. Some of them are known to influence cerebral hemodynamics (i.e., fentanyl,^63^^–^^65^ midazolam,^66^ remifentanil,^67^ atropine sulfate,^68^ and lidocaine^69^). Our study is not designed to investigate these interactions and it is not trivial to consider these effects since they are not well known. Since the primary goal of the study the direct comparison of the optical and BIS signals, we have decided to remove the duration of their administration from all the signals as described in the following section.

### 2.3 Data Evaluation

The data were first evaluated by fitting the respective semi-infinite medium solutions of the photon diffusion equation to the optical measurements.^70^^–^^72^ The fitting was done using the downhill-simplex or Nelder-Mead method in MATLAB™’s implemented fminsearch function.^73^^,^^74^ Further data analysis was then carried out in R^75^ (v4.0.1, R Core Team, 2019) and the integrated development environment RStudio (v1.1.5042, RStudio, Inc., Boston), especially for the calculation of OEF and ${\mathrm{CMRO}}_{2}$,^70^ artifacts removal and data re-sampling. Data collected during patient movements in the surgery were excluded from the dataset. When further medications clearly changed the optical signals or BIS, they were generally recorded with a mark and were excluded as well during post-processing. The data were grouped in 5 s bins for each patient to match the BIS sample size.

The oxy and deoxyhemoglobin (${\mathrm{HbO}}_{2}$ and Hhb) contents were calculated using the wavelength-dependent absorption coefficients ($\mu_{a}$) from TRS measurements (see Sec. 2.1). Three main tissue constituents (${\mathrm{HbO}}_{2}$, Hhb, and $H_{2}O$) were considered. The tissue oxygen saturation (${\mathrm{StO}}_{2}$) was calculated as the ratio of ${\mathrm{HbO}}_{2}$ concentration over the sum of ${\mathrm{HbO}}_{2}$ and Hhb concentrations.

Assuming that blood volume percentage in the venous compartment (γ)^76^ does not change ($\gamma_{1}=\gamma_{2}$), the measures of ${\mathrm{StO}}_{2}$ were converted into relative OEF (rOEF) and ${\mathrm{CMRO}}_{2}$ (${\mathrm{rCMRO}}_{2}$) changes using the relation:^48^^,^^70^^,^^76^^–^^78^

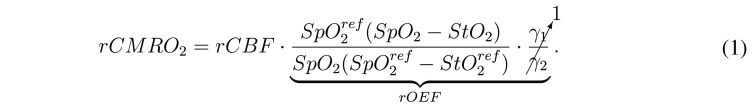
where the superscript *ref* stays for reference and is generally chosen during a steady state such as the baseline of measurement. In the present study, two different references were used, as it is clarified in the followings.

### 2.4 Statistical Analysis

The hypotheses of this study were: (1) after propofol induction, CBF and ${\mathrm{CMRO}}_{2}$ decrease (as BIS does) with respect to the initial baseline; (2) after propofol reduction, CBF and ${\mathrm{CMRO}}_{2}$ increase (as BIS does); (3) during the beginning of the surgery, the time of the changes in CBF, OEF and ${\mathrm{CMRO}}_{2}$ shows a correlation with the times of change of BIS; and and (4) during the end of the measurement, the time of the changes in CBF, OEF, and ${\mathrm{CMRO}}_{2}$ shows a correlation with the times of change of BIS.

Relevant R packages exploited for this purpose and data visualization were *nlme* (v. 3.1-152), *lme4* (v. 1.1-27), *lmerTest* (v. 3.1-3), and *stats* (v. 3.6.3).

First, the times corresponding to the induction of TIVA and the end of propofol infusion were identified for all measurements on BIS time-traces according to the marked events.

Short periods (100 s) around these times were selected for each measurement session as including the LOC and ROC. A total of four windows of interest were then defined during these two periods, two for the initial period and two for the final period. All windows had a duration of 100 s and they were chosen and labeled as follows. The “before anesthesia onset” window was defined just before the event of propofol injection. The before anesthesia onset was the baseline for the initial period and thus used to normalize all data for the entire initial period, providing relative values ($r{\mathrm{BIS}}_{i}$ where the index “i” stays for induction). The “after stabilization” window following the anesthetics induction was symmetrically centered around a point that was automatically identified as the minimum value after BIS decreased at least below 40 and over a range of the signal that included the arrival at a stable condition after LOC. The “recovery” window (recovery and used as a second baseline) was selected after the maximum point that was automatically identified after BIS increased at least up to 65. The recovery window was the baseline for the second period and was used to normalize all, providing relative values ($r{\mathrm{BIS}}_{r}$ where “r” stays for recovery). The “before end of anesthesia” window was taken just before the event marking the end of propofol infusion. The choice of using two baselines was made to focus just on the initial and final parts separately, discarding all the changes that occurred during the whole surgery and artifacts. The same four temporal windows were then applied to the optically derived relative time-traces ($r{\mathrm{CBF}}_{i}$, $r{\mathrm{CBF}}_{r}$, $r{\mathrm{OEF}}_{i}$, $r{\mathrm{OEF}}_{r}$, $r{\mathrm{CMRO}}_{2i}$, and $r{\mathrm{CMRO}}_{2r}$).

Afterward, the median of all relative time-traces was calculated separately over the windows and divided into four groups according to the window of origin (before anesthesia onset, after stabilization, before end of anesthesia, and recovery). Medians were chosen over means to account for any remaining outliers that may influence the mean. Boxplots for each group of medians and each variable were then depicted for data visualization purposes. To test hypotheses, Wilcoxon paired samples tests (Wilcoxon signed-rank test) were used to compare groups two by two: data for groups after stabilization were then compared to the data for group before anesthesia onset, while before end of anesthesia to recovery. For ${\mathrm{rBIS}}_{i}$, ${\mathrm{rCBF}}_{i}$, ${\mathrm{rOEF}}_{i}$, and ${\mathrm{rCMRO}}_{2}i$, it was tested the alternative that the after stabilization group was lower than the baseline group before anesthesia onset. On the contrary, ${\mathrm{rBIS}}_{r}$, ${\mathrm{rCBF}}_{r}$, and ${\mathrm{rCMRO}}_{2}r$ were tested for the alternative that the recovery group was greater than the baseline group before end of anesthesia; while for ${\mathrm{rOEF}}_{r}$ it was tested if the recovery group was lower than the before end of anesthesia one. A similar approach was taken also with the other physiological variables (MAP, HR, and ${\mathrm{SpO}}_{2}$), as explained in Appendix 6.2.

Furthermore, a percentage of change was calculated to quantify the amount of change per each time-trace with respect to the baseline. This percentage was expressed as the ratio between the absolute value of the difference of the median value of the group after stabilization or recovery and the median value during the baseline before anesthesia onset or recovery respectively multiplied by 100 and the median of the corresponding baseline period again. Similarly were calculated their respective interquartile ranges (IQR) as a percentage.

Then, the time-traces per each measurement session were considered separately, aiming at testing the same hypotheses on an individual basis. Unpaired Wilcoxon test was used to test whether the difference between the window of after stabilization or before end of anesthesia and the mean of their respective baseline window before anesthesia onset or recovery was different from zero. The test was repeated for both single-sided cases, greater or lower than zero. A change was considered significant if the p-value was lower than 0.05. When the difference was significantly greater than zero, it was acknowledged as a positive change, an increase; on the contrary, as a negative change, a decrease. If none of the tests was significant, it was marked as constant, “no change.” Whenever a change was found to be significant, its direction, either as an increase or a decrease with respect to baseline, was counted and the prevalent direction for the non-invasive signals compared to the BIS one.

Finally, the correlation in time between non-invasive hemodynamic profiles and the BIS was investigated by verifying whether they were subject to concomitant changes. In this regard, the times at which the time-traces for each measurement session exhibited a change were identified. The initial period focused on the data around the LOC, starting 100 s before the TIVA induction and lasting until a new stable level of the time-trace was reached. The final period was selected starting 100 s before the end of propofol infusion and lasting until the end of the measurement session.

**Fig. 2:**
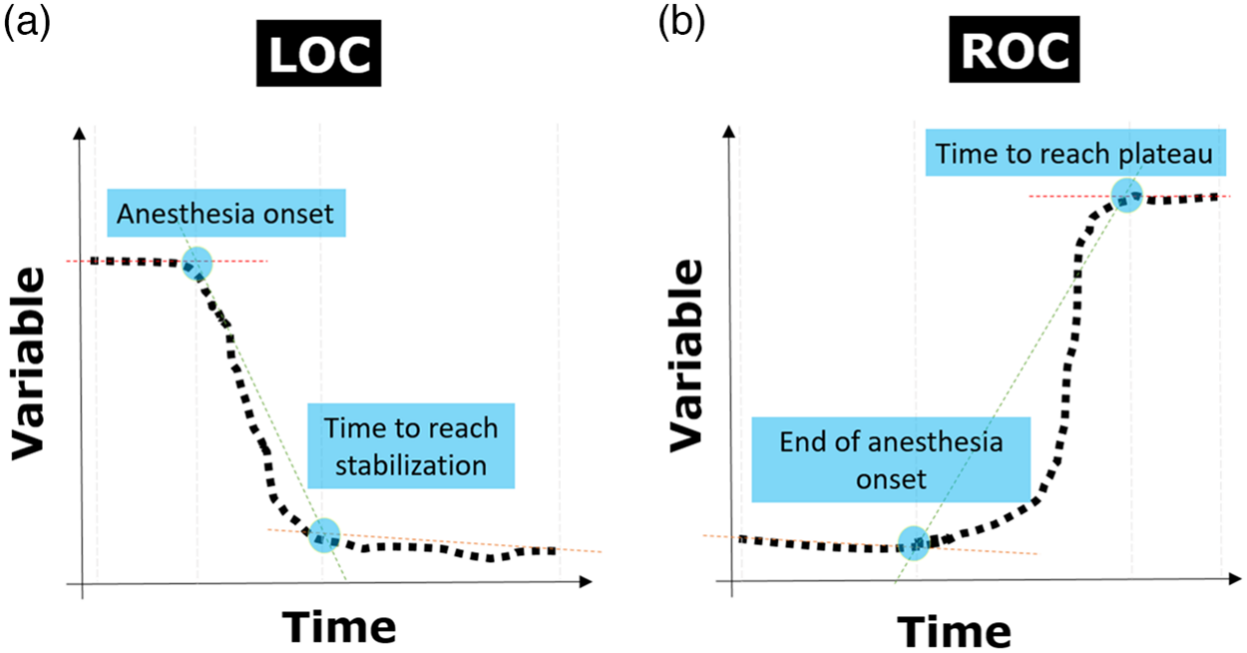
Exemplification of the automatic segmentation by the Lavielle method. (a) The period of LOC for the variables of relevance (${\mathrm{rBIS}}_{i}$, ${\mathrm{OEF}}_{i}$, ${\mathrm{CBF}}_{i}$, and ${\mathrm{CMRO}}_{2i}$) in time is depicted. The period includes an initial baseline, a transition period and then another period that reaches a stable condition. The moments in the time axis where the segments split the time-traces are then collected. In the LOC, the anesthesia onset corresponds to the change point between the initial baseline and the beginning of the transition; while the time to reach stabilization coincides with the moment in which the transition ends and the new stability level is reached. (b) Similarly, on the right there is an example focused on the ROC. Here, the end of anesthesia onset and time to reach plateau are identified.

The identification of the moments of change was performed automatically by exploiting the Lavielle method^79^^,^^80^ (function *lavielle* in R), which is capable of detecting the moment when a statistical change occurs in a time-dependent signal and is exemplified in Fig. 2. The number of segments into which the algorithm divides a time-trace (either initial or final) was set to three, to match the expected three periods, i.e., baseline, transition, and new stable level. As a consequence, two points of change were identified for the initial period and two for the final one. They were labeled as “anesthesia onset” and “time to reach stabilization” for the initial transition, while as “end of anesthesia onset” and “time to reach plateau” for the waking up transition.

The four points were collected for the four variables of interest (rBIS, rCBF, rOEF, and ${\mathrm{rCMRO}}_{2}$) per measurement session and grouped according to the four labeled times. Some cases were discarded, as detailed in Appendix 6.1. Then, each group of points was used to check the correlation in time between rBIS points and the optically related ones using R Pearson correlation coefficients, which were computed and reported together with p-values (significant when lower than 0.05). Additionally, to visualize the relation, rBIS was plotted versus rCBF, rBIS versus rOEF and rBIS versus ${\mathrm{rCMRO}}_{2}$ and linear regression lines were depicted with the 95% confidence interval.

## 3 Results

### 3.1 Study Population

About 29 patients were enrolled in this study, but six had to be excluded from the analysis because of missed recording of the propofol induction and final recovery. Details about data unavailability and discarded subjects are reported in Appendix 6.1. About 23 patients (15 men and 8 women) were included for further analysis. For this population, the average (standard deviation) age was 55.2(11) years and the body mass index (BMI) was $28.5(4)\;\mathrm{Kg}/m^{2}$. Demographic and clinical data are provided in Table 1.

**Table 1:** Demographic table related to the included subjects and reporting the sex (M: male, F: female), age, BMI, and surgery type. The discarded subjects were not included in this table, and for this reason, the identification (ID) numbers of the subjects are not sequential.

ID	Sex	Age (years)	BMI ($\mathrm{Kg}/m^{2}$)	Surgery type
1	M	62	27.7	Posterior spinal fusion
2	M	57	26.1	Retrogasserian neurotomy
3	F	62	33.9	Lumbar discectomy
5	F	73	24	Lumbar laminectomy
6	F	39	26.2	Discectomy and fixation of first cervical vertebra
7	M	65	26.4	Fixation of first cervical vertebra
8	M	39	21.8	Lumbar disc hernia (relapsed)
9	M	41	32.7	Arthrodesis
10	F	59	33.3	Morbid obesity
11	M	60	29.4	Cholecystectomy
12	F	56	34.9	Cholecystectomy
13	M	48	34	Thyroidectomy
16	M	47	29.3	Hemithyroidectomy
17	M	54	28.1	Exploratory laparoscopy
18	F	37	21.5	Cholecystectomy
22	M	45	25	Hemithyroidectomy
23	M	73	24.1	Cholecystectomy
24	F	68	30.2	Cholecystectomy
25	F	71	28.1	Hemicolectomy
26	M	65	26.6	Cervical laminectomy
27	M	41	27.4	Cervical corpectomy
28	M	59	27.5	Laminectomy
29	M	48	37	Cervical discectomy

### 3.2 Optical Data

**Fig. 3:**
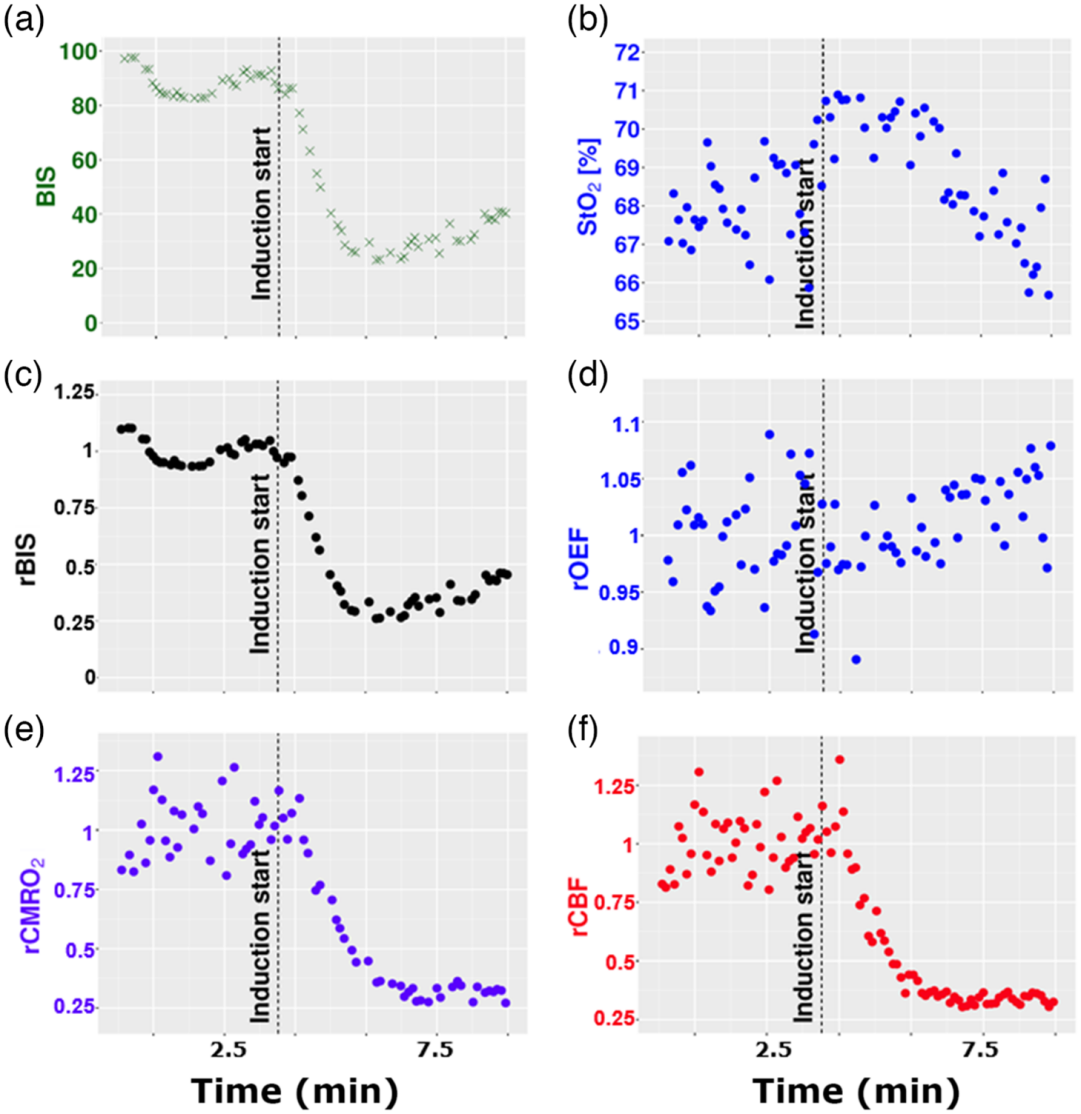
Representative time evolution of the (a) BIS; (b) tissue oxygen saturation (${\mathrm{StO}}_{2}$); (c) rBIS. (d) The relative oxygen extraction fraction (rOEF); (e) the relative cerebral metabolic rate of oxygen extraction change (${\mathrm{rCMRO}}_{2}$); and (f) the relative CBF change (rCBF) during anesthesia induction ($t_{0}$ 3 minutes) by propofol (1%).

**Fig. 4:**
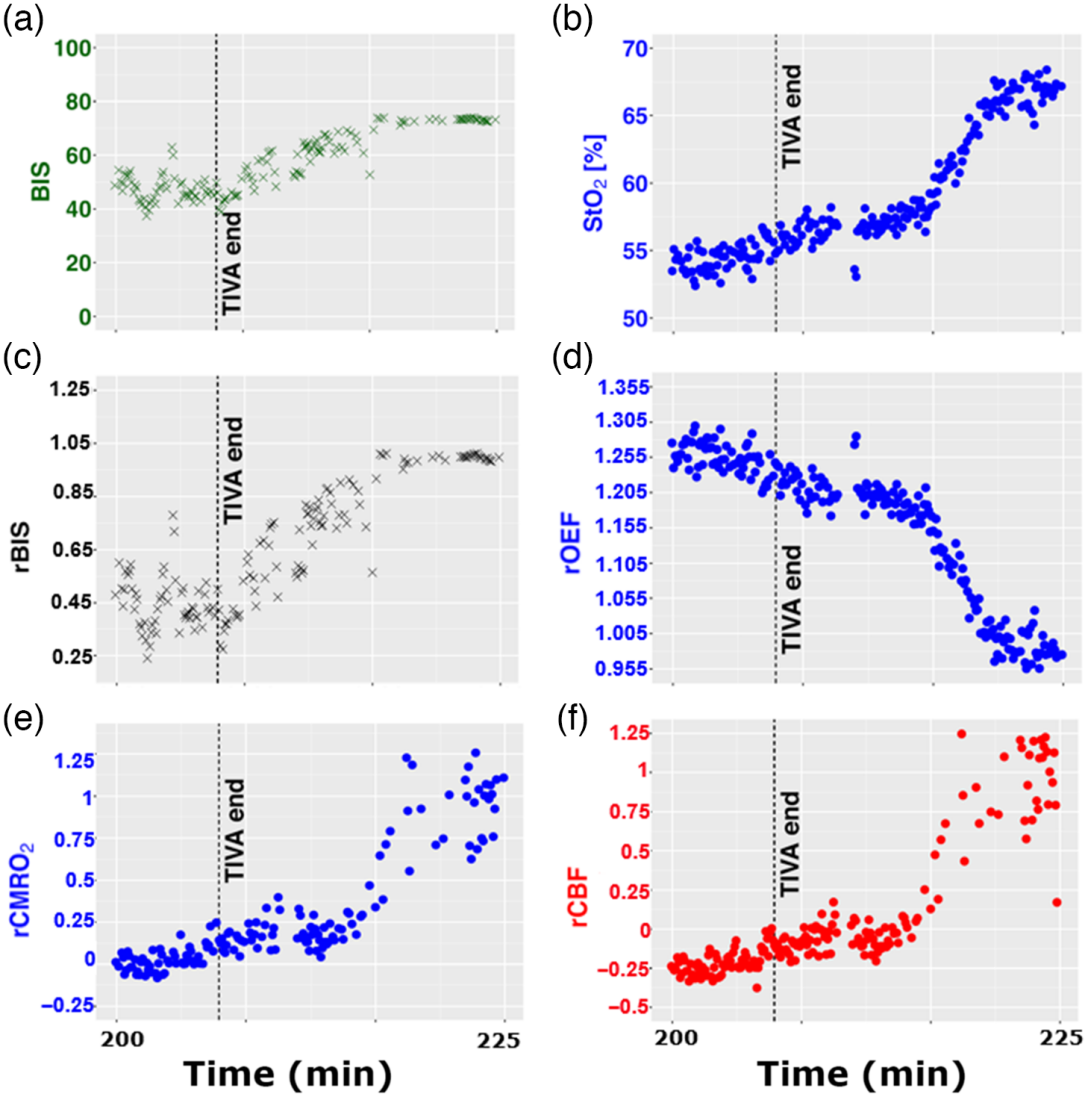
Representative time evolution during ROC of the (a) BIS; (b) tissue oxygen saturation (${\mathrm{StO}}_{2}$); and (c) relative BIS (rBIS). The relative changes of (d) the oxygen extraction fraction (rOEF), e) the cerebral metabolic rate of oxygen extraction (${\mathrm{rCMRO}}_{2}$) and (f) the CBF (rCBF) are also shown at the end of the surgery performed under TIVA with propofol (1%) ($t_{\text{end}}$ 207 minutes).

Figures 3 and 4 show examples of time-traces during anesthesia induction and over the wake-up phase. The relative changes in BIS, OEF, ${\mathrm{CMRO}}_{2}$, and CBF from the left cerebral hemisphere are represented as well as absolute ${\mathrm{StO}}_{2}$ and BIS.

In Fig. 3, the above-mentioned parameters are plotted around the initiation of general anesthesia by TIVA of propofol at $t_{0}$ 3 minutes and are excerpts from the entire procedure from subject 13.

In this example, the BIS value decreases from around 90 to about 30 [Fig. 3(a)], which represents a characteristic BIS drop due to induction of general anesthesia by TIVA with propofol. The ${\mathrm{StO}}_{2}$ starts from around 68%, rises to 71% and then decreases to 66%. rBIS has a drop of 66% [Fig. 3(c)] if the baseline value is considered as 100%. Relative OEF shows an upward tendency after induction. Similarly to the observed BIS change, the ${\mathrm{rCMRO}}_{2}$ and the rCBF [Fig. 3(f)] decreased by about 60% [Fig. 3(e)] referred to a 100% baseline. The baseline relative values for the optical variables were equal to 1 (100%). After the change, during the selected window that was used for the following analysis, the relative values of the median (standard deviation) were: 0.29(0.03) for the rBIS, 0.35(0.05) for rCBF, 0.32(0.05) for rCMRO_2_ and 0.92 (0.09) for rOEF.

In Fig. 4, a characteristic time evolution of BIS, ${\mathrm{StO}}_{2}$, rBIS, rOEF, ${\mathrm{rCMRO}}_{2}$, and rCBF are plotted during the ROC phase of subject 29. The propofol perfusion was stopped at $t_{\text{end}}$ 207 minutes.

BIS increases from around 40 to 50 to about 70 as a reaction to the end of the general anesthesia by TIVA with propofol [Fig. 4(a)]. ${\mathrm{StO}}_{2}$ increases from around 55% up to 67% [Fig. 4(b)]. In this case, a window after the TIVA end is averaged and defined as a new reference as previously described. An rBIS raise of about 55% is observed [Fig. 4(c)] if the new reference is set as 100%. ${\mathrm{StO}}_{2}$ increases, and since the ${\mathrm{SpO}}_{2}$ did not show a change for this subject, rOEF decreased by about 27% [Fig. 4(d)]. ${\mathrm{rCMRO}}_{2}$ and rCBF showed a positive change recovering of up to 100%.

**Fig. 5:**
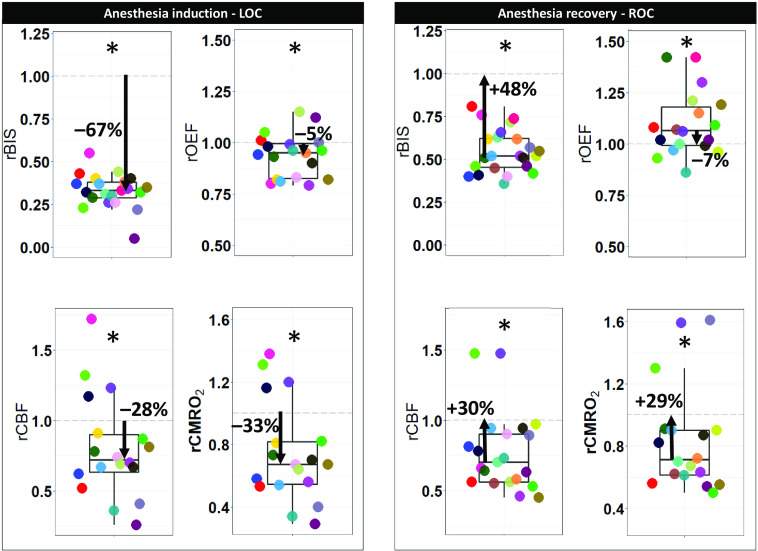
Data group visualization by boxplots for the periods of LOC and ROC. Both periods include the data distributions for the four variables (rBIS, rCBF, rOEF, and ${\mathrm{rCMRO}}_{2}$) around the group median and are all compared to their respective baseline period (either before anesthesia onset or recovery), which equals one and is represented by a dashed gray line. Percent changes of the medians are also reported close to arrows that show the direction (higher-up or lower-down) with respect to baseline. P-values for testing the difference of the group median with respect to baseline are reported. Asterisks indicate significant p-values. Subjects are color-coded in the same way for all variables.

In Fig. 5, on the left, the results of the group analysis are reported. For the initial part of the measurement, focused on the LOC, ${\mathrm{rBIS}}_{i}$, ${\mathrm{rCMRO}}_{2i}$, ${\mathrm{rCBF}}_{i}$, and ${\mathrm{rOEF}}_{i}$ all showed a significant decrease for the corresponding after stabilization window with respect to their before anesthesia onset (p<0.0001, p<0.01, p<0.01, and p<0.01, respectively). The amount of percentage change calculated considering the baseline as 100% was −67% (IQR−62% to −71%) for ${\mathrm{rBIS}}_{i}$, −33% (IQR−18% to −46%) for ${\mathrm{rCMRO}}_{2i}$, −28% (IQR−10% to −37%) for ${\mathrm{rCBF}}_{i}$ and −5% (IQR−1% to −18%) for ${\mathrm{rOEF}}_{i}$.

Similarly, the group results for the final part regarding the ROC phase are on the right part of Fig. 5. ${\mathrm{rBIS}}_{r}$, ${\mathrm{rCMRO}}_{2r}$, ${\mathrm{rCBF}}_{r}$, and ${\mathrm{rOEF}}_{r}$ all showed a significant change for the corresponding before end of anesthesia window with respect to the recovery window (p<0.0001, p<0.05, p<0.01, and p<0.001, respectively). This change was an increase for the first three variables, with a percentage of +48%(IQR +38% to +55%) for ${\mathrm{rBIS}}_{r}$, +29%(IQR +17% to +39%) for ${\mathrm{rCMRO}}_{2r}$, and +30% (IQR +10% to +44%) for ${\mathrm{rCBF}}_{r}$. Instead, ${\mathrm{rOEF}}_{r}$ decreased by −7%(IQR −20% to −1%) during the final period.

We did not find any significant change in the other monitored systemic parameters.

The tests results of the subject-by-subject analysis of the direction of change are summarized in Table 2. For ${\mathrm{rBIS}}_{i}$, all after stabilization to before anesthesia onset comparisons lead to a significant decrease (21/21). For ${\mathrm{rCBF}}_{i}$, 14/18 comparisons led to a significant decrease, while 4/18 to a significant increase. For ${\mathrm{rCMRO}}_{2i}$, after stabilization windows showed a significant decrease with respect to before anesthesia onset for 12/18 measurements, a significant increase in 4/18 and showed no change in 2/18 cases. ${\mathrm{rOEF}}_{i}$ decreased significantly in 10/19 comparisons, increased significantly in 3/19 and showed no change in 6/19.

**Table 2:** Summary of single subjects results. For all variables, the number of events was counted and reported against the total number of measurements included for that subject. For the initial part, related to the LOC, after stabilization-mean(before anesthesia onset) windows were tested against zero; while for the final part, related to the ROC, before end of anesthesia-mean(recovery) windows were tested against zero. For the beginning, the majority of the cases for all variables present a significant decrease. Instead, rCBF, ${\mathrm{rCMRO}}_{2}$, and rBIS show a majority of increases for the final part, while rOEF an increase.

	Induction - LOC				Recovery - ROC			
	CBF	OEF	${\mathrm{CMRO}}_{2}$	BIS	CBF	OEF	${\mathrm{CMRO}}_{2}$	BIS
**Significant increase**	4/18	3/19	4/18	0/21	19/21	5/20	13/19	23/23
**Non-significant change**	0/18	6/19	2/18	0/21	0/21	4/20	2/19	0/23
**Significant decrease**	14/18	10/19	12/18	21/21	2/21	10/20	3/19	0/23

The evaluation of the variables during the ROC showed that ${\mathrm{rBIS}}_{r}$ had a significant increase for all measurements (23/23). rCBFr had a significant increase in 19/21 cases and a significant decrease in 19/21. ${\mathrm{rCMRO}}_{2r}$ decreased significantly in 13/18, stayed constant in 2/18 and increased in 3/18 subjects. ${\mathrm{rOEF}}_{r}$ increased in 5/19 measurements, did not vary significantly in 4/19, while it decreased significantly in 10/19.

**Fig. 6:**
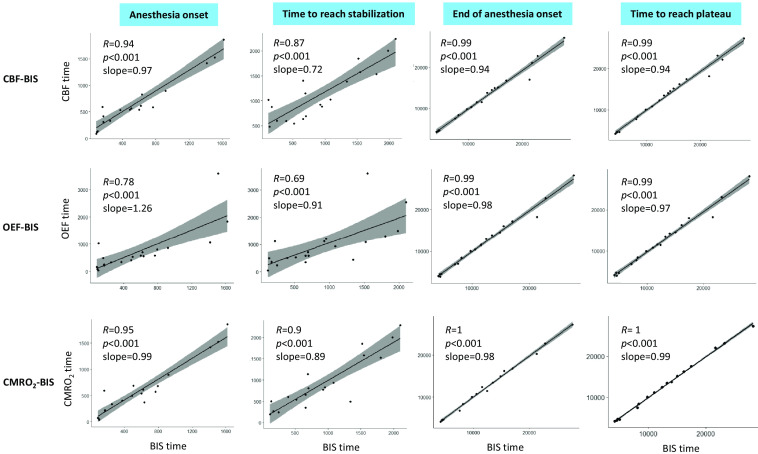
Linear regression plots for the four automatically retrieved time points identifying a change in the time-traces for all four variables of interest. BIS time is plotted versus CBF time, then versus OEF time and finally versus ${\mathrm{CMRO}}_{2}$ time. Pearson correlation coefficients R and p-values are reported for each plot. Gray areas represent a 95% confidence interval built around the regression line.

In relation to the analysis of the correlation in time, a total of 12 plots with the linear regressions and R Pearson correlation values are shown in Fig. 6. The results for the initial period are reported in the first two columns of plots: all cases showed a p-value lower than 0.05. For the anesthesia onset, Pearson coefficients R were 0.94 for CBF-BIS time, R=0.78 for OEF-BIS time and R=0.95 for ${\mathrm{CMRO}}_{2}$-BIS time. For the time to reach stabilization, R was equal to 0.87 for CBF-BIS time, R=0.69 for OEF-BIS time and R=0.9 for ${\mathrm{CMRO}}_{2}$-BIS time. R is always higher than 0.6 and positive. For the final part of the surgery, all cases showed a p-value lower than 0.05 and are reported in the third and fourth columns in Fig. 6. For the end of anesthesia onset, Pearson coefficients R were 0.99 for CBF-BIS time, R=0.99 for OEF-BIS time and R=1 for ${\mathrm{CMRO}}_{2}$-BIS time. For the time to reach plateau, R was equal to 0.99 for CBF-BIS time, R=0.99 for OEF-BIS time and R=1 for ${\mathrm{CMRO}}_{2}$-BIS time.

## 4 Discussion

Propofol, a γ-Aminobutyric acid (GABA) receptor agonist, is a potent intravenous hypnotic drug used for induction and maintenance of sedation and general anesthesia.^81^^–^^83^ Due to propofol’s neurophysiological effects, such as decreased CBF,^82^ intracranial pressure, and cerebral metabolic rate, and its potential influence on the dynamic and static autoregulation,^84^ there is a need for continuous, intra-operative, cerebral monitoring. Currently, online monitoring is utilized to ensure an appropriate loss of consciousness during surgery.

Here, we have argued the need for the monitoring of cerebral oxygen metabolism and evaluated a near-infrared, diffuse optical monitor as a potential candidate. Particularly, we have shown that, as expected, propofol promoted a change in the state of consciousness as reflected in both the BIS and ${\mathrm{CMRO}}_{2}$ readings. We have demonstrated the feasibility of the method by measuring the cerebral metabolism during propofol induction and maintenance of anesthesia and described its impact in 23 patients, over a range of eighteen different types of surgeries. We now discuss how our findings compare and relate to the previous literature.

In the past, several studies have exploited NIRS to monitor the cerebral oxygenation over a broad variety of surgery types.^18^^,^^85^^–^^90^ However, these studies focused either on pre- and post-surgery assessments^90^ or on the effect of the induction of anesthesia while focusing on various procedures.^91^^–^^95^ One recent study by Zavriyev et al.,^96^ using both NIRS (a frequency domain system) and DCS, was employed during surgery in anesthetized patients but did not include LOC and ROC periods. In their study, the authors have concluded that this type of platform is effective in optimizing cerebral perfusion by comparing the CBF changes during hypothermic circulatory arrest alone and with retrograde or with antegrade cerebral perfusion. This supports our claim that hybrid methods can have a role during operations.

Other studies employed standard NIRS quantifying oxyhemoglobin and deoxyhemoglobin concentrations which is difficult to quantitatively compare with our study.^17^^,^^93^^,^^97^ For example, Liang et al.^97^ employed a research device based on continuous-wave NIRS to measure the propofol induced changes during LOC and ROC, but only reported values related to changes in ${\mathrm{HbO}}_{2}$, Hhb, THC, and one parameter related to the cardiac pulsation in arbitrary units. Therefore, only the trends are actually comparable to our findings. During LOC, ${\mathrm{HbO}}_{2}$ increased significantly, Hhb significantly increased, THC significantly increased, and the cardiac pulsation-related parameter significantly decreased. On the contrary, during ROC, ${\mathrm{HbO}}_{2}$ decreased significantly, Hhb significantly decreased, THC significantly decreased, and the cardiac pulsation-related parameter significantly increased. Overall, if we base an ${\mathrm{StO}}_{2}$ calculation on the median values of ${\mathrm{HbO}}_{2}$ and Hhb reported in the paper, we could speculate that, for their group (N=11), ${\mathrm{StO}}_{2}$ decreased during LOC, implying an increase in OEF, and an increase during ROC, suggesting a decrease in OEF. These are in qualitative agreement with our findings.

The topic was reviewed by Hernandez-Meza et al.,^10^ showing ${\mathrm{HbO}}_{2}$, Hhb, THC, and ${\mathrm{StO}}_{2}$ changes as measured by continuous-wave NIRS after propofol induction. In particular, ${\mathrm{StO}}_{2}$ increased after LOC (again by interpreting the trend results from oxy and deoxyhemoglobin concentrations), which usually corresponds to a decrease in OEF, although it was not explicitly calculated. Due to this, one could only speculate that the results of the trends for the optical signals are in agreement with the findings of our paper and are opposite with respect to the previous article cited.

Similar results during LOC were reported by others. Park et al.^16^ reported that ${\mathrm{StO}}_{2}$ increased by 5% to 6%, Taskaldiran et al. reported an ${\mathrm{StO}}_{2}$ increase of 4%,^98^ Valencia et al. 9% increase,^44^ and Kim et al., 10% increase.^99^ Conversely, an ${\mathrm{StO}}_{2}$ decrease of 2% to 5% was reported by others,^100^^,^^101^ which would lead to an increase in OEF. In summary, the previous literature based on clinical NIRS have not provided a conclusive result quantifying the cerebral oxygenation changes during LOC and ROC phases.^17^^,^^102^ This may be a physiological issue (different procedures), a device/algorithm complication and/or an example of the complex coupling between CBF and cerebral blood oxygenation.

As these studies highlight, our study is more comprehensive and unique compared to the previous ones. We have measured continuously during the entire surgical procedure, and focused on the moments of largest change at the cerebral and metabolic levels. We note that another option would have been to measure a surrogate of local CBF using transcranial Doppler ultrasound but that technology has been deemed impractical for routinely monitoring in the surgical environment.^103^ In the study by Manquat et al.,^103^ this was attempted by the authors alongside NIRS monitoring, with the overarching aim to assess the cerebral autoregulation of the patients. Unfortunately, only baseline values of the blood flow velocity and ${\mathrm{StO}}_{2}$ were reported in the paper and are, therefore, not comparable to our findings.

Overall, other modalities were also utilized for cerebral monitoring and imaging. For instance, O15-labeled water, oxygen, and carbon monoxide positron emission tomography was used to evaluate the changes after propofol induction.^4^ rOEF increased by 15% after LOC, ${\mathrm{rCMRO}}_{2}$ decreased by about 38% and rCBF decreased by about 43%. The decrease in ${\mathrm{rCMRO}}_{2}$ and rCBF are in agreement with our findings at the group level. On the contrary, in our case OEF for the group decreased by 5% and had an opposite direction of change. In another study, the authors found a significant CBF decrease by about 5 ml/100 g/min after mild propofol sedation by MRI arterial spin labeling,^104^ which is also in line with our findings for the LOC period.

In our study, the group analysis involving the periods of the time-traces around LOC highlighted a significant decrease in ${\mathrm{rBIS}}_{i}$, ${\mathrm{rCBF}}_{i}$, ${\mathrm{rOEF}}_{i}$, and ${\mathrm{rCMRO}}_{2i}$ for the LOC. Both variables that represent a surrogate of metabolism (electrophysiology versus hemodynamics based) showed that the cerebral oxygen metabolism was, as expected, decreased. The relative (percent) change estimated by ${\mathrm{rCMRO}}_{2i}$ was approximately half of the electrophysiological estimate (${\mathrm{rBIS}}_{i}$). This could highlight, once more, that BIS is a complex, compound index that was developed to measure the level of consciousness rather than the cerebral oxygen metabolism.^20^^,^^21^ If confirmed, a direct measure of oxygen metabolism by optics could be included in the relevant clinical algorithms.

We have also found a significant increase for ${\mathrm{rBIS}}_{r}$, ${\mathrm{rCBF}}_{r}$, and ${\mathrm{rCMRO}}_{2r}$ for the ROC during the group analysis, while ${\mathrm{rOEF}}_{r}$ had a significant decrease. The sources of the literature regarding the ROC are very scarce in the NIRS panorama, which does not allow us to provide an effective comparison with previous studies. In general, these findings confirm the expected effect of propofol anesthesia on both CBF and ${\mathrm{CMRO}}_{2}$, which maintain their coupling during the LOC and ROC phases, and follow the same trend as BIS as expected by the literature.^4^^,^^69^^,^^105^ Some examples of the correlation between rCBF and rBIS can be found in Refs. 4, 6, 7, and 106. Collectively, these observations corroborate the ability of non-invasive diffuse optical techniques to measure an index of cerebral oxygen metabolism.

Moreover, we also note, as visible in Fig. 5, the high variability in the response of the subjects in both LOC and ROC group analysis. This highlights one of our motivations for this study, i.e., the need for online monitoring for personalized management of propofol dosage. To further examine this, we have carried out a subject-by-subject evaluation that was summarized in Table 2 gives The majority of the changes in CBF and ${\mathrm{CMRO}}_{2}$ occurred in the same direction as BIS. Interestingly, OEF had a more heterogeneous response among subjects which could explain the discrepancies in NIRS results which can only estimate OEF. We can speculate that one reason for this paradoxical OEF response can be the loss of autoregulation or being in a state close to hypoxia. This needs to be confirmed in future studies. However, other studies concluded that propofol itself does not affect the cerebral autoregulation.^84^ In the present study, we could not evaluate autoregulation because the study was not designed for this purpose, had a poor temporal resolution and inadequate synchronization between neuromonitoring and blood pressure data.

However, other studies concluded that propofol itself does not affect the cerebral autoregulation.^84^ In the present study, we could not evaluate autoregulation because the study was not designed for this purpose, had a poor temporal resolution and inadequate synchronization between neuromonitoring and blood pressure data.

Another possible speculation to explain the variability encountered in the response of the optical data could be the fact that it is known that surgical procedures that involved laparoscopy (i.e., exploratory laparoscopy, cholecystectomy, hemithyroidectomy, etc.) may suffer from additional physiological changes to the cerebral hemodynamics and metabolism that can be confounders to the propofol induced changes. In fact, laparoscopy can provoke compression on the inferior vena cava due to air inflation in the abdomen, which may affect the central venous pressure, and therefore, the venous CBF and volume.^58^ However, the general trend is maintained in agreement to the overall results. A larger sample size and a study statistically powered for this additional hypothesis is needed to further analyze this point.

Since this observation was made, one could consider evaluating whether we have observed potential states of misery perfusion, which is a condition in which the brain enters whenever the demand for oxygen and energy is higher than the supply that the blood can provide.^107^^–^^109^ Misery perfusion risk is deemed to be high whenever there is a concomitant decrease in rCBF with an increase in rOEF. We have carried out a basic analysis (data not shown) and checked each subject to highlight possible ischemia risk. No cases were found. This concept was also investigated by our group in a different cohort of patients.^110^ This is a topic for future investigation.

Finally, we have demonstrated the timing of the changes in hemodynamic and oxygen metabolic variables and those of BIS were closely correlated [R>0.69 for the LOC (p<0.001), R=0.99 (p<0.001) for the ROC]. These results are in agreement with the hypothesis that the propofol effect on the brain does not decouple oxygen metabolism and electrophysiology in general.

We highlight that both diffuse optical modalities in our study are more advanced than the commonly utilized clinical cerebral oxymeters.^111^^,^^112^ These systems, such as the INVOS (Somanetics), EQUANOX (Nonin), NIRO (Hamamatsu Photonics, Japan), or OM (Shimadzu Co., Japan) monitors, are based on continuous wave NIRS and are used for the assessment of the risk of hypoxia in the clinics.^111^^,^^113^ They have been employed for example during surgery (i.e. aortic surgery, carotid endarterectomy, and cardiac surgery), stroke, in neonatology, in the investigation of the cognitive and visual functions and in traumatic brain injury monitoring.^86^^,^^112^^–^^117^ INVOS devices were also used for brain monitoring during general anesthesia and are compatible with BIS.^86^^,^^115^ Unfortunately, due to the physics of the problem, continuous wave NIRS is best suited for trend monitoring and is reliable only during selected, homogeneous populations/procedures such as during hypothermia treatments or pediatric cardiac surgery.^86^ They also can not estimate the delivery of oxygen, i.e., cannot measure CBF. Using a hybrid system,^70^ we are able to quantify CBF.

The use of TRS in our study improves the separation of extracerebral (e.g., scalp and skull) signals from intracerebral ones which is prohibitively difficult in compact CW NIRS systems.^49^^,^^118^ Furthermore, TRS allows the separation of absorption effects from scattering effects and the measurement of absolute values.^114^ We note that while DCS is a CW method, due to the large blood flow differential between scalp/skull and cerebral tissue, DCS is approximately three times more sensitive to the intracerebral signals.^47^^,^^119^ Moreover, newer devices based on these techniques are also capable of more precise synchronization as well as higher sampling rate, that allow one to obtain better insight into physiological mechanisms and additional information, such as pulsatility index and cerebral autoregulation.^120^^–^^123^

However, this separation of intracerebral and extracerebral signals is a matter of ongoing research and it has to be further tested. In the estimation of ${\mathrm{CMRO}}_{2}$, based on optical data, this becomes even more complex since it combines data from two different modalities (TRS and DCS). The resulting partial volume effects can lead to underestimation of rOEF and rCBF.^86^^,^^124^^–^^131^ Since ${\mathrm{rCMRO}}_{2}$ is derived from these quantities [see Eq. (1)], the possible underestimation translates to the calculated metabolism in a complex manner. Nonetheless, several studies making use of TRS on the healthy subjects showed consistency among the retrieved values even compared to two-layered model approaches.^132^^–^^134^ Therefore, the current approach can be considered a sufficient trade-off for the evaluation of the changes in the optical properties in this validation study. As the field matures, more realistic analysis methods could be widely adopted. In literature, TRS methods were proved to intrinsically provide deeper tissue information than CW-based ones.^49^^,^^111^^,^^135^ We also acknowledge that nowadays two-layers analysis or moments-based methods can be used to ensure deeper tissue information assessment.^136^^,^^137^

The need for ${\mathrm{CMRO}}_{2}$ is an important, yet an unsettled question. This paper does not claim to show the benefits of ${\mathrm{CMRO}}_{2}$ measurements. Instead, it uses this particular protocol to investigate the relationship between BIS and ${\mathrm{CMRO}}_{2}$. The benefits would have to be investigated in a study with an outcome variable, such as safety (is metabolism reduced too much?) or depth of awareness (are people really unconscious or will develop post-operative delirium?). A head-to-head study of different methods based on an outcome variable is hopefully motivated by our results.

Further research could be carried out to characterize the response observed in the optical data to the most common additional drugs and medications that are administered during surgery. If this reveals clear patterns, it could then be included as a refinement of the interpretation of the optical data, hence driving the future potential implementation of online optical data corrections similar to the ones that the BIS algorithms appear to possess. For example, it has been reported that BIS has internal data corrections artifact removal including those due to additional medications, which has strengthened the monotonic dose-response relationship.^14^ A future study looking at this could be designed based on the data provided in this manuscript to achieve proper power and to guide the data recording.

In the present study, relative values were used for two main reasons: (1) so far, intraoperative physiological changes are considered more clinically relevant than absolute values and current CW-NIRS monitors display percent changes with respect to a baseline^18^^,^^138^ and (2) intrasurgical artifacts were quite relevant in this study and this lead to focusing on the two periods of interest (LOC and ROC) each with its own baseline. However, obtaining absolute values in commonly utilized units is also important although challenging for the current state of the art, especially for DCS. Previous studies have reported successful methods for the conversion of BFI units provided by DCS into common units (ml/100/min) in Refs. 139–141, which may be used after further confirmation. With better instrumentation and probes, this could be implemented for intra-operative monitoring, which is the topic of ongoing research and development.

## 5 Conclusion

Overall, this study illustrated the ability of hybrid DO to simultaneously evaluate cerebral oxygen metabolism and an index of the level of consciousness during propofol-induced anesthesia for surgery. The level of heterogeneity of the observed response highlighted the need for personalized monitoring for efficient management of the patient’s level of consciousness and also for ensuring the safety of the brain. These results motivate future research and development and larger clinical studies.

## 6 Appendix

### 6.1 Discarded and Unavailable Data Details

As reported in Sec. 3, some subjects were excluded from further analysis. Six patients had to be excluded because the recording of the LOC and ROC were missed. About 23 measurements were included.

Unfortunately, some more time-traces were partially discarded, since the quality of the data was not sufficient, especially for TRS data.

For TRS, one quantitative criteria for rejection was the signal-to-noise ratio (SNR) calculated as the ratio between the maximum value of the light pulse and the standard deviation taken within a portion of the signal prior to the pulse, the background. The threshold set was SNR<10. Qualitatively, the shape of the TRS laser pulse was also considered as an important feature to reject data.

For DCS, a minimum of 10 kHz for the count-rate was set as a threshold for rejection. The main cause of poor data quality or its deterioration was postural changes during surgery, which led to a loosening of the probe. Over time, the quality generally suffered from this and data points were lost towards the end of the measurements. The main consequence is reflected in the higher exclusion rate for the ROC periods. Often, data rejection corresponded to marked events over time and notes were inspected to verify their origin. The repercussions of the removal on the data were different for the different moments of the analysis. The numbers of available measurements evaluated in each analysis are reported in the following Table 3.

**Table 3:** Details of the number (N) of measurements included for each analysis type according to the period (LOC or ROC) and by physiological signal ($N_{\mathrm{BIS}}$, $N_{\mathrm{CBF}}$, $N_{\mathrm{OEF}}$, and $N_{{\mathrm{CMRO}}_{2}}$).

Analysis	Period	$N_{\mathrm{BIS}}$	$N_{\mathrm{CBF}}$	$N_{\mathrm{OEF}}$	$N_{{\mathrm{CMRO}}_{2}}$
Group	LOC	21	19	20	19
	ROC	23	21	21	20
Subject-by-subject	LOC	21	19	20	19
	ROC	21	19	19	18
Segmentation	LOC	21	20	21	20
	ROC	23	21	18	17

### 6.2 Additional Physiological Variables

The monitored systemic parameters were ${\mathrm{SpO}}_{2}$, HR, and MAP. The successful measurements with valid recordings were for the LOC: N MAP = 12, N HR = 21, and N ${\mathrm{SpO}}_{2}=23$; while for the ROC: N MAP = 10, N HR = 18, and N ${\mathrm{SpO}}_{2}=23$. For most of the measurements, in fact, we had no data points and most of the times this was due to a failure in the recordings after patient movement.

For these variables, we have carried out a group analysis in the same fashion as BIS and the optical parameters, meaning that we have taken the median values over the same windows and built a dataset for each variable, separating LOC and ROC. Then, we tested with the same Wilcoxon test, to check whether the distributions between the values post-induction or pre-recovery were different from their baseline.

In summary, during LOC we did not find any significant difference (LOC: p-value MAP = 0.07, p-value ${\mathrm{SpO}}_{2}=0.8$, and p-value HR = 0.6) between the groups. Instead, during the ROC, we found that the median value after recovery was significantly higher for HR (p=0.004) and MAP (p=0.02), while no significant change for ${\mathrm{SpO}}_{2}$ (p=0.48).
